# Supplementary material for: Acoustic modulation of mechanosensitive genes and adipocyte differentiation
Source: Commun Biol. 2025 Apr 16;8:595. doi: 10.1038/s42003-025-07969-1 (PMC12003795; doi:10.1038/s42003-025-07969-1)
Supplement: Supplementary file 2 — Supplementary information [file 42003_2025_7969_MOESM2_ESM.pdf]

# Supplementary Figures and Legends

# Acoustic modulation of mechanosensitive genes and adipocyte differentiation

**Masahiro Kumeta[1,2]*, Makoto Otani[3], Masahiro Toyoda[4], Shige H. Yoshimura[1,2]**

[1] Graduate School of Biostudies, Kyoto University, Kyoto, Japan

[2] Center for Living Systems Information Science (CeLiSIS), Kyoto University, Kyoto, Japan

[3] Graduate School of Engineering, Kyoto University, Kyoto, Japan

[4] Faculty of Environmental and Urban Engineering, Kansai University, Osaka, Japan

*Corresponding author: kumeta@lif.kyoto-u.ac.jp (MK)

**Supplementary Fig. 1**

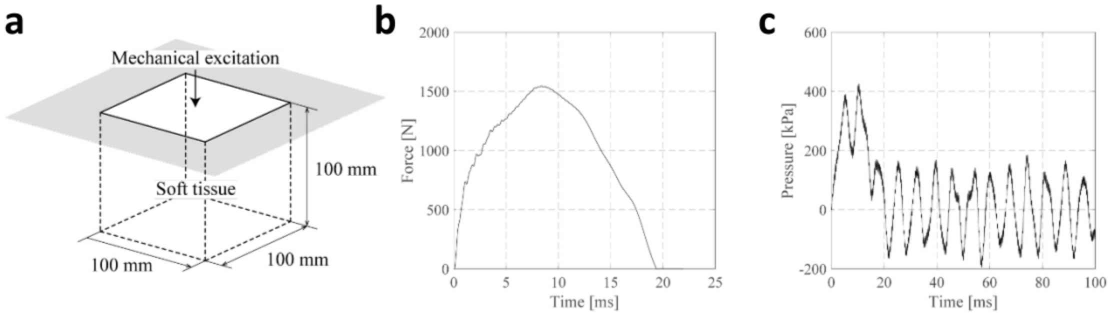


**Supplementary Fig. 1 | Simulation analysis of sound transmission in an elastic object via physical contact.**

**a)** An elastic object of 0.001 $m^3$ volume with a $1 \times 10^5$ $N/m^2$ shear elastic constant was subjected to physical contact with an impact ball (YI-01, Rion). Analysis was performed based on the vibroacoustic finite-difference time-domain method. **b)** Properties of the input force applied to the top of the object via physical contact. **c)** Properties of the vibroacoustic pressure detected at the centre of the object. As the simulation condition did not consider energy dissipation, circulation of pressure was observed. The first pressure peak indicates that the maximum pressure owing to this physical contact was ~400 kPa.

**Supplementary Fig. 2**

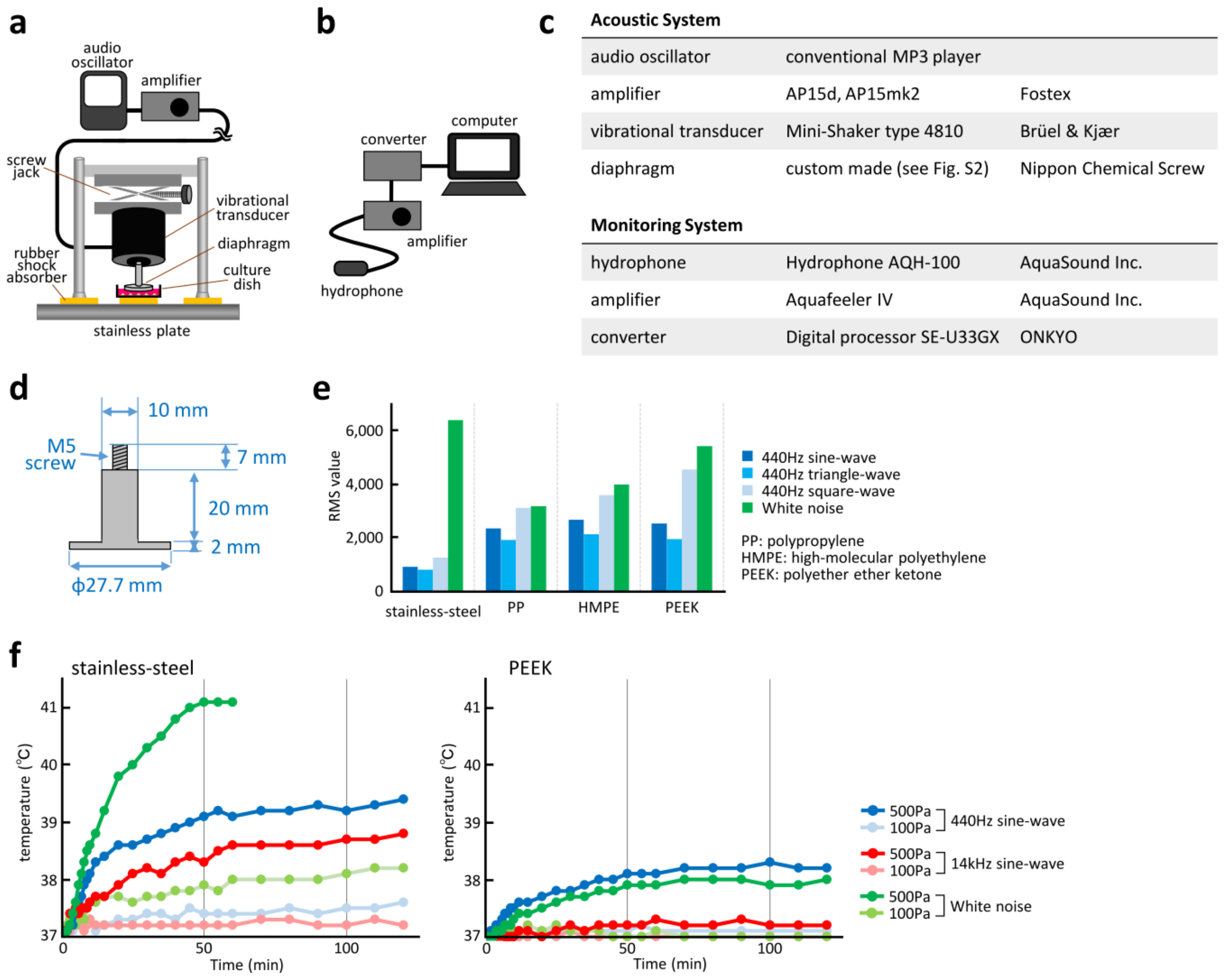


**Supplementary Fig. 2 | Setup of the acoustic irradiation and monitoring systems.**
**a)** Schematic illustration of the direct sound emission system used in this study. The vibrational transducer was attached to a small screw jack and fixed to a metal shelf. A custom-made diaphragm was attached to the transducer. The system and dish were put on a 5-mm-thick 450 × 450 mm stainless plate through rubber shock absorbers to cancel vibrations and avoid slipping during acoustic emission. The tip of the diaphragm was attached to the culture medium by adjusting the screw jack. The sound signal was output using a conventional MP3 player through an amplifier. **b)** Schematic illustration of the sound monitoring system. A hydrophone was placed in a water tank to monitor the intensity of sound transmitted into the culture medium. The details of the sound intensity measurement procedure are described in the Methods. **c)** Equipment used in the acoustic and monitoring systems. **d)** Design of custom-made diaphragm. Head was made of a 2-mm-thick plate with diameter of 27.7 mm, designed to cover 80% of the 30 mm dish (actual diameter: 31 mm). The tail contained an M5 screw attached to a vibrational transducer. **e)** The diaphragm was made of four different materials: stainless-steel, polypropylene (PP), high-molecular polyethylene (HMPE), and polyether ether ketone (PEEK). The 440 Hz sine-wave, triangle-wave, square-wave, and white noise sound was emitted at the same output

level, and the sound intensity was measured in water. RMS values show that the stainless-steel diaphragm exhibits the highest intensity when emitting white noise, whereas the PEEK diaphragm shows a high and constant output. **f)** Changes in temperature owing to acoustic stimulation. Stainless-steel and PEEK diaphragms were used to emit acoustic signals in a 30 mm dish containing 3 ml of water; 440 Hz sine-wave, 14 kHz sine-wave, and white noise sound was output at 500 Pa and 100 Pa for 2 h. Time-lapse monitoring of the temperature clearly shows the high thermal conductivity of the stainless-steel diaphragm, which transferred heat from the vibrational transducer to the sample. The temperature changes using the PEEK diaphragm were smaller; in particular, increases in temperature with 100 Pa output were limited within 0.2 ℃. Based on these findings, the PEEK diaphragm was selected as the standard.

## Supplementary Fig. 3

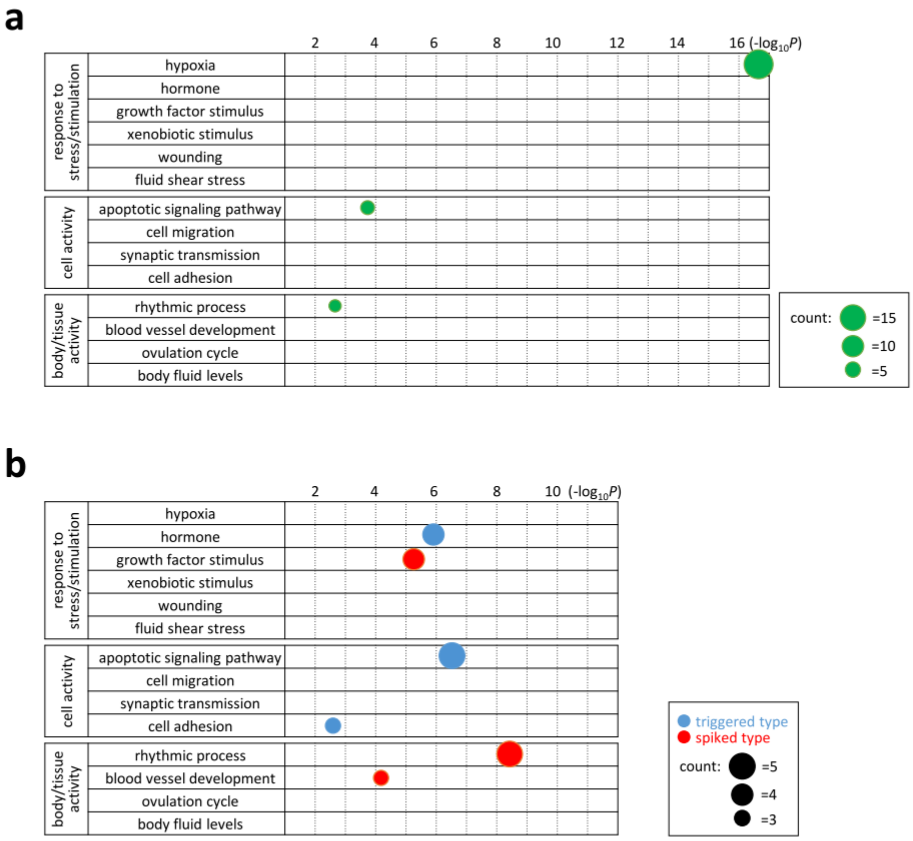


**Supplementary Fig. 3 | Gene annotation analyses of specific groups of sound-sensitive genes.** **a)** Annotation analysis of genes upregulated by 440 Hz stimulation and downregulated by 14 kHz sound, indicated in red in Fig. 1f. **b)** Annotation analysis of trigger-type (blue) and spike-type (red) upregulated genes presented in Fig. 2a.

**Supplementary Fig. 4**

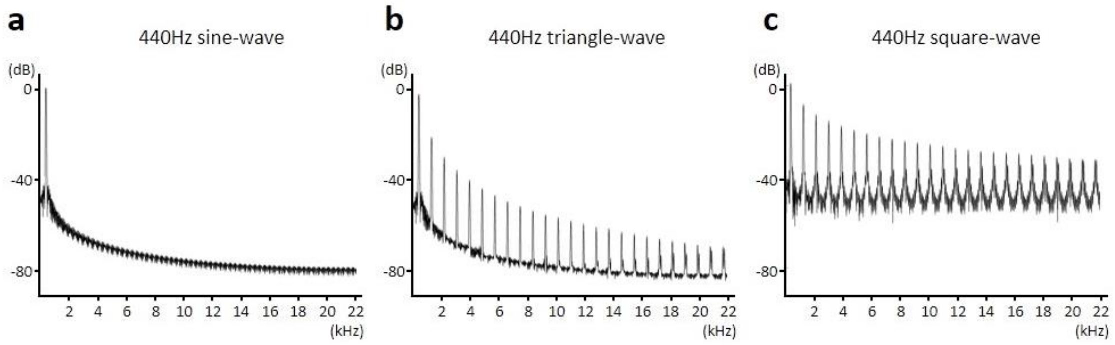


**Supplementary Fig. 4 | Frequency characteristics of sine-, triangle-, and square-waves.** The averaged spectrum of 440 Hz for different waveforms was obtained using a Fourier transform. Results of **a)** sine-wave, **b)** triangle-wave, and **c)** square-wave signals are shown.

## Supplementary Fig. 5

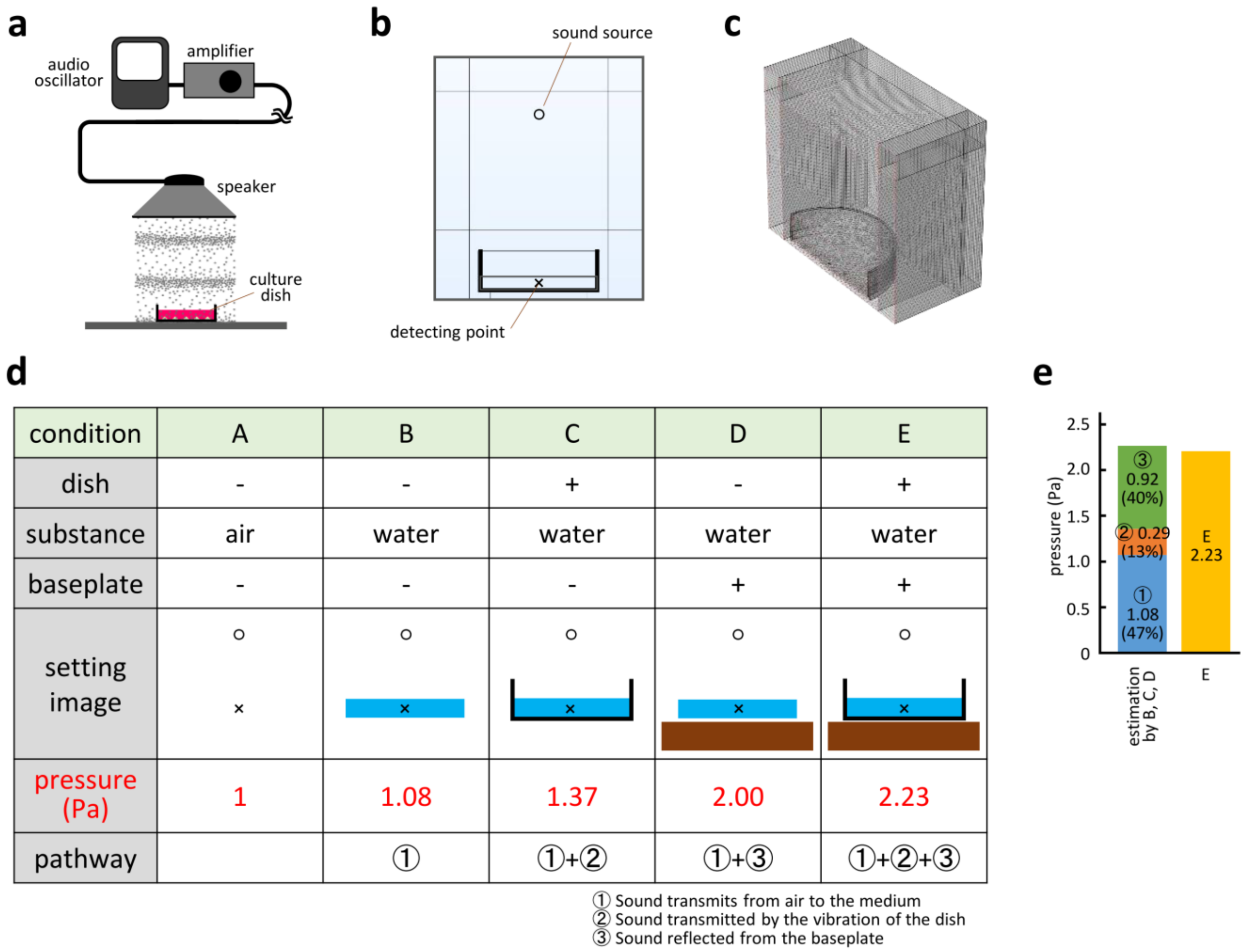


**Supplementary Fig. 5 | Simulation analyses of acoustic propagation using the indirect sound emission system.**

**a)** Schematic illustration of indirect sound emission system used in a previous study[12]. A full-range loudspeaker was set above the culture dish to emit sound, as described in the Methods. **b)** Simulation field indicating the sound source, detection point, and substances such as water and dishes. **c)** 3D image of the simulated acoustic field. The dissected image is presented, showing the sound source, detection point, and dish in the field. **d)** Pressure levels detected using different settings. The pressure level simulated in air (A) was set as the standard, and the same sound source was used. Pressures propagated by the sound transmitted from air to the medium (①), transmitted by the vibration of the dish (②), and reflected from the baseplate (③) were estimated by comparing conditions B, C, and D. **e)** The sum of the pressures ①, ②, and ③ was relatively equal to the pressure detected under condition E, demonstrating that cells received only 40% of the direct transmitted sound and approximately 60% of the reflected/re-irradiated sound that might be altered.

**Supplementary Fig. 6**

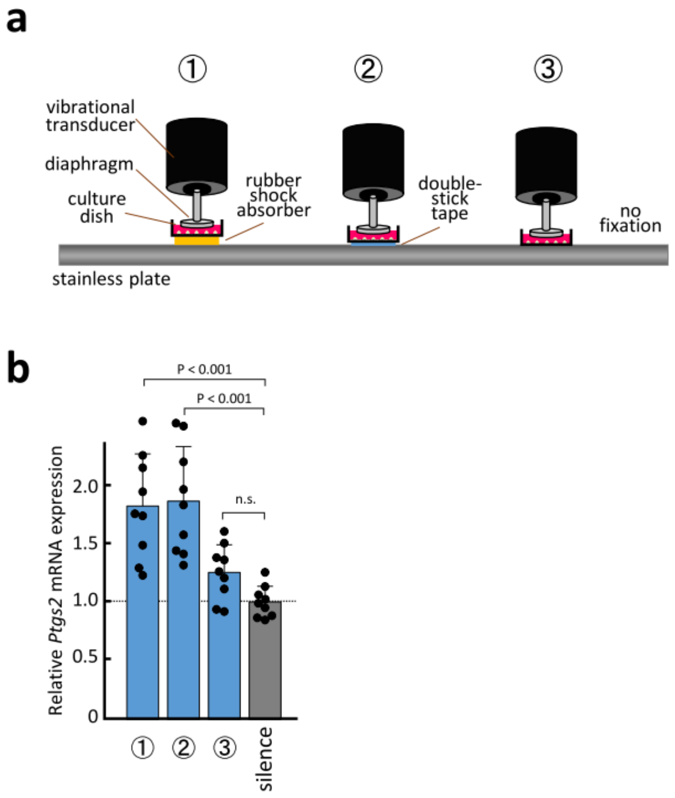


**Supplementary Fig. 6 | Effect of dish vibration on the acoustic response of *Ptgs2*.**
**a)** Schematic illustration of the direct sound emission system used to observe the effect of dish vibrations on gene regulation. Standard dish setting (①), dish firmly attached to the plate by a double-stick tape (②), and dish without fixation (③) were prepared. After 2 h of emission of 440 Hz sine-wave sound at 100 Pa, total RNA was extracted, and *Ptgs2* expression was measured using qPCR analysis. **b)** qPCR results clearly demonstrate that the vibration of the dish abolished the effect of directly emitted sound, suggesting an opposing effect of indirectly transmitted sound on sound-sensitive gene responses. Bars represent +SD from three biological replicates of three independent experiments, and statistical significance was evaluated using one-way ANOVA followed by Tukey's HSD test.

**Supplementary Fig. 7**

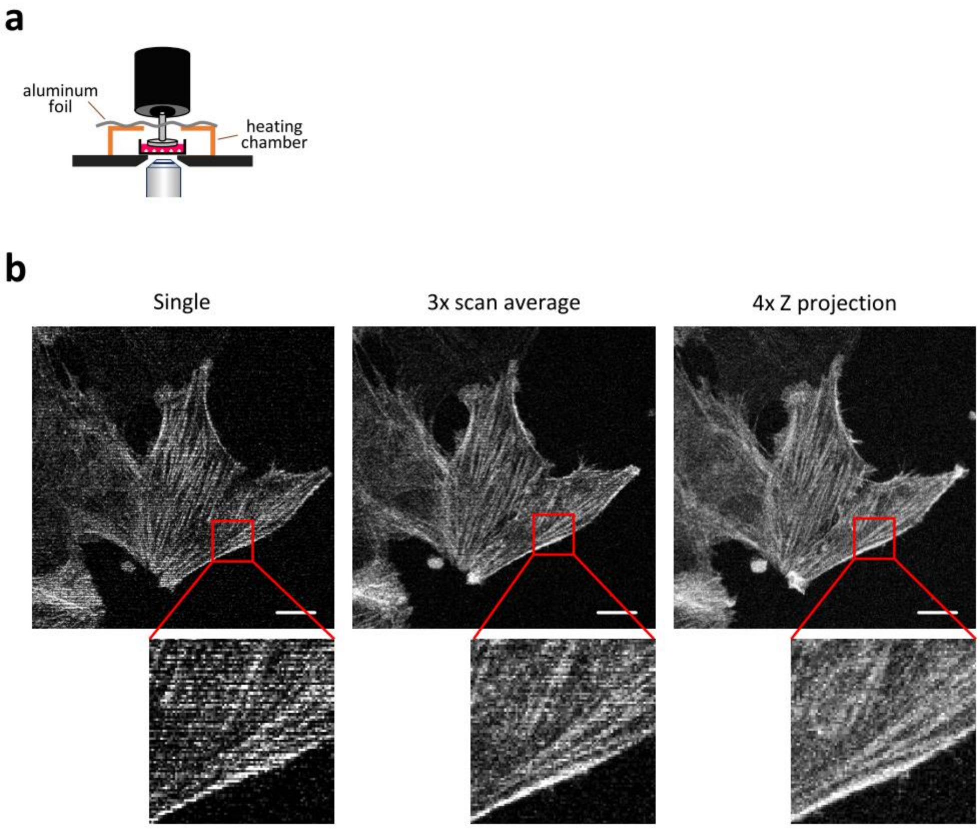


**Supplementary Fig. 7 | Image-processing procedure for live cell observation under acoustic stimulation.**

**a)** Schematic illustration of the direct sound emission system at the microscope stage. Cells transfected with EGFP-Lifeact were grown in a glass-bottomed dish and observed under a confocal laser-scanning microscope. The diaphragm was inserted through a hole in the lid of the heating chamber and covered with aluminum foil to stabilise the temperature. Images were captured every minute for 1 h. **b)** Image-processing procedure. Sound at 440 Hz emitted at 100 Pa disturbed the laser scanning image acquisition and generated scratching noise in the single-plane image. To cancel this noise, data from three scans were averaged on a single plane, and four differently focused images with 1 µm Z-scale intervals were projected.

**Supplementary Fig. 8**

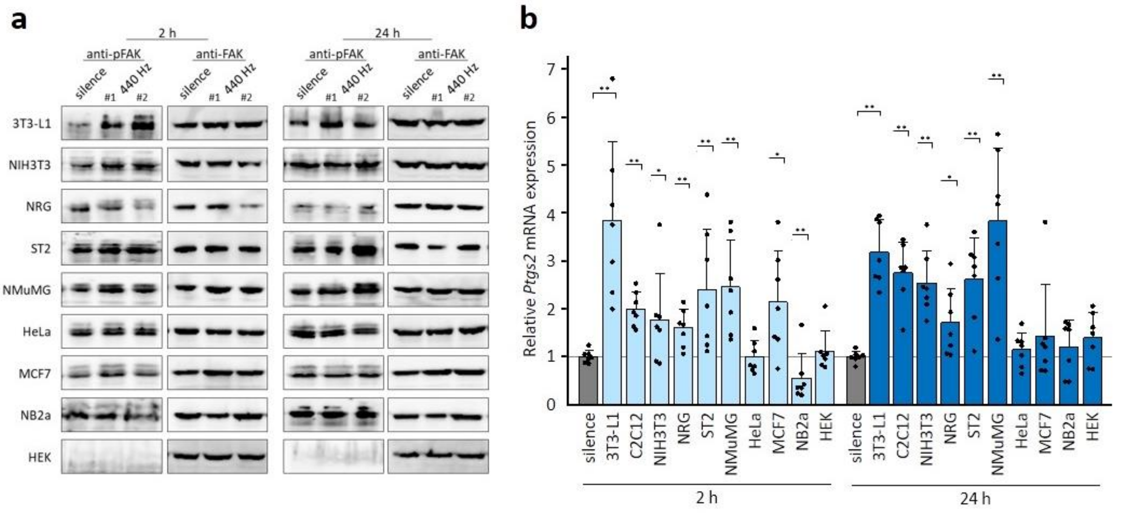


**Supplementary Fig. 8 | Responses of FAK phosphorylation and *Ptgs2* gene expression to acoustic stimulation in different cell lines.**

**a)** 3T3-L1, HEK, HeLa, MCF7, NB2a, NIH3T3, NMuMG, NRG, ST2 cells were subjected to 440 Hz acoustic stimulation at 100 Pa intensity for 2 and 24 h. Phosphorylation states of FAK Y397 was analysed by quantifying pFAK/FAK signal intensities of Western blotting using anti-FAK and anti-pFAK antibodies and the effect of acoustic stimulation was evaluated over silent condition. Results of two biological replicates (#1 and #2) are shown. **b)** qPCR analysis of *Ptgs2* expression in different cell lines. Cells were subjected to acoustic stimulation by 440 Hz sine-wave sound at 100 Pa for 2 h and 24 h. The results clearly demonstrate a significant upregulation of *Ptgs2* expression following acoustic stimulation. Bars represent +SD from three biological replicates of two independent experiments, and statistical significance was evaluated using Tukey's multiple comparison test against silent condition. *: P<0.5, **: P<0.1.

**Supplementary Fig. 9**

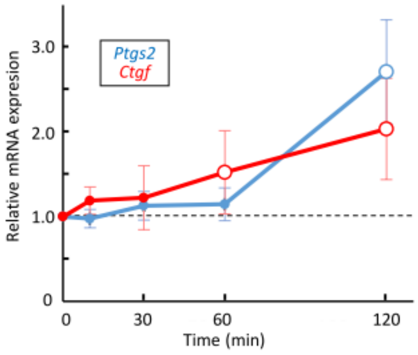


**Supplementary Fig. 9 | Sound-sensitive gene responses in shorter time points.** Expression levels of *Ptgs2* (blue) and *Ctgf* (red) in C2C12 cells treated with 440 Hz sound at 100 Pa for 10, 30, 60 and 120 minutes. Bars represent ±SD from 3 biological replicates of 2 independent experiments, and statistical significance was evaluated using Welch's t-test. Open circles indicate statistical significance of $P < 0.05$ compared with the silent sample.

**Supplementary Fig. 10**

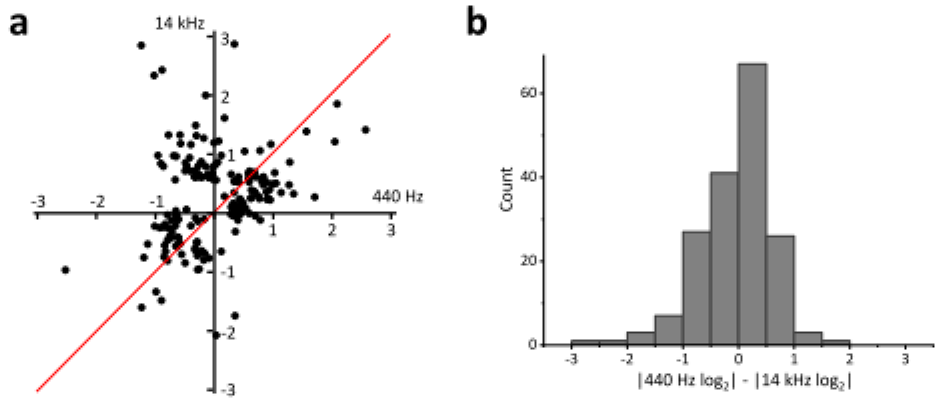


**Supplementary Fig. 10 | Correlation of gene responses against 440 Hz and 14 kHz acoustic stimulation.**

**a)** Correlation of log2 fold differences of sound-sensitive gene responses after 2 and 24 of 440 Hz (X-axis) and 14 kHz (Y-axis) acoustic stimulation. Red line indicates X = Y. 54.5% of the genes were X > Y, while 45.5% were X < Y. **b)** Histogram of the differences between 440 Hz and 14 kHz gene responses.

**Supplementary Fig. 11**

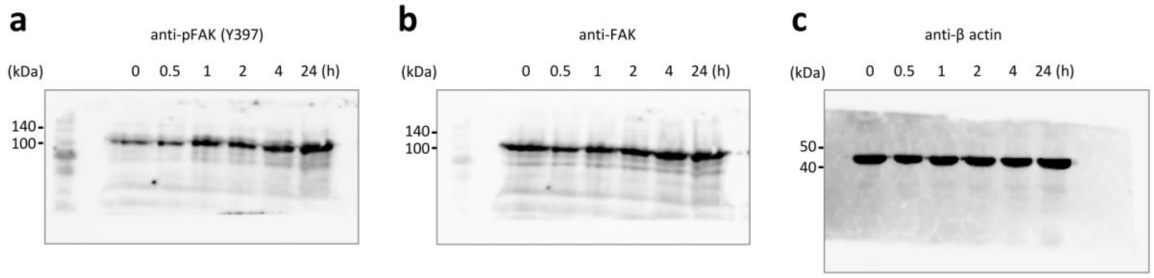


**Supplementary Fig. 11 | Unedited images of western blots using anti-pFAK, FAK, and β-actin.** Unedited images of data shown in Fig. 3d. Western blot analysis of C2C12 whole-cell lysate taken at the indicated time point after starting 440 Hz continuous acoustic stimulation at 100 Pa. After blotting with an anti-phospho Y397 FAK antibody **(a)**, the membrane was stripped and reblotted with an anti-FAK antibody **(b)**. Anti-β actin blotting was performed as a loading control **(c)**.

**Supplementary Movie 1:** Live cell observation of C2C12 cells under acoustic stimulation. C2C12 cells expressing EGFP-Lifeact were observed under acoustic stimulation with 440 Hz sine-wave sound at 100 Pa. Images were acquired every minutes for 60 min. Image processing procedures are described in Supplementary Fig. 7.

**Supplementary Movie 2:** Live cell images of expanded and retracted areas of C2C12 cells under acoustic stimulation. Based on the data presented in Supplementary Movie 1, expanded and retracted areas in 1 min were shown in green and red, respectively.

**Supplementary Movie 3:** Live cell observation of C2C12 cells in silent condition. C2C12 cells expressing EGFP-Lifeact were observed under silent condition.

**Supplementary Movie 4:** Live cell images of expanded and retracted areas of C2C12 cells in silent condition. Based on the data presented in Supplementary Movie 3, expanded and retracted areas in 1 min were shown in green and red, respectively.

**Supplementary Audio 1: 440 Hz Sine Wave (60s)**
**Supplementary Audio 2: 14k Hz Sine Wave (60s)**
**Supplementary Audio 3: White Noise (60s)**
**Supplementary Audio 4: 440 Hz Triangle Wave (60s)**
**Supplementary Audio 5: 440 Hz Square Wave (60s)**
