# Supplementary material for: Acoustic modulation of mechanosensitive genes and adipocyte differentiation
Source: Commun Biol. 2025 Apr 16;8:595. doi: 10.1038/s42003-025-07969-1 (PMC12003795; doi:10.1038/s42003-025-07969-1)
Supplement: Supplementary file 3 — Description of Additional Supplementary Files [file 42003_2025_7969_MOESM3_ESM.pdf]

Description of Additional Supplementary Files

File Name: Supplementary Data
Description: The source data behind the graphs in the paper.

File Name: Supplementary Movie 1
Description: Live cell observation of C2C12 cells under acoustic stimulation.

File Name: Supplementary Movie 2
Description: Live cell images of expanded and retracted areas of C2C12 cells under acoustic stimulation.

File Name: Supplementary Movie 3
Description: Live cell observation of C2C12 cells in silent condition.

File Name: Supplementary Movie 4
Description: Live cell images of expanded and retracted areas of C2C12 cells in silent condition.

File Name: Supplementary Audio 1
Description: 440 Hz Sine Wave (60s).

File Name: Supplementary Audio 2
Description: 14k Hz Sine Wave (60s).

File Name: Supplementary Audio 3
Description: White Noise (60s).

File Name: Supplementary Audio 4
Description: 440 Hz Triangle Wave (60s).

File Name: Supplementary Audio 5
Description: 440 Hz Square Wave (60s).
